# Allergen specific immunotherapy has no influence on standard chemistry and hematology laboratory parameters in clinical studies

**DOI:** 10.1186/2045-7022-4-18

**Published:** 2014-05-22

**Authors:** Dietrich Häfner, Viola Gödicke, Annemie Narkus

**Affiliations:** 1 Allergopharma GmbH & Co. KG, Hermann-Körner-Str. 52, 21465 Reinbek, Germany

**Keywords:** Allergen immunotherapy, Clinical chemistry and hematology

## Abstract

**Background:**

A set of standard clinical chemistry and hematology parameters are usually measured during clinical studies. The major outcome of these standard tests is to control that the drug investigated does not lead to pathophysiological changes in respective organs or blood. In some cases based on scientific rationale such tests may not be needed.

In this paper we report on a standard set of clinical chemistry and hematology laboratory parameters measured before and after treatment in three different immunotherapy studies, representing different routes of administration and different formulations.

**Methods:**

Thirteen hematological laboratory parameters and eight clinical chemistry parameters were evaluated from three double-blind, placebo-controlled, randomized, multi-centre, phase III studies. The three studies include one with sublingual immunotherapy (n = 185), one subcutaneous immunotherapy trial with an aluminium hydroxide-adsorbed recombinant hypoallergenic Bet v1-FV (n = 211) and one with pre-seasonal subcutaneous immunotherapy with a 6-grass pollen allergoid (n = 154).

**Results:**

Allergen specific immunotherapy with both administration forms and formulations respectively did not show any influence on any of the 21 laboratory parameters analyzed. Few patients had a change in laboratory parameters from within normal range at baseline to either below or above at end-of-treatment. No differences between active and placebo were seen with respect to number of patients with such a change.

**Conclusions:**

This study with different preparations and routes of application indicates that the value of repeated measurements of standard clinical chemistry and hematology parameters during allergen immunotherapy should be discussed further.

## Background

As a routine analysis for evaluation of safety in clinical development programs for new drugs, a set of standard clinical chemistry and hematology parameters are measured. The major outcome of these standard tests is to control that the drug investigated does not lead to pathophysiological changes in respective organs or blood. Guidelines from EMA on safety pharmacology studies discuss such a pharmacology core battery for investigation of vital functions and that in some cases based on scientific rationale such tests may not be needed [1]. In the present paper we focus on clinical chemistry and hematology variables, only.

Allergen specific immunotherapy is known to be an efficient treatment for respiratory allergic diseases and based on clinical investigations also shown to have a good safety profile including only potential allergic side effects in patients already allergic to the allergen they are treated with [2]. In recent years new administration forms and formulations of allergens are being developed [3] and many studies are ongoing and being planned. The recent guidelines from EMA on clinical development of allergen products for allergen specific immunotherapy discuss safety parameters which should be addressed such as routine laboratory hematology, biochemical tests and urinalysis [4].

In this paper we report on a standard set of clinical chemistry and hematology laboratory tests measured before and after treatment in three different immunotherapy studies, representing different routes of administration and different formulations.

## Methods

Thirteen hematological laboratory parameters and eight clinical chemistry parameters (Table 1) were evaluated from three double-blind, placebo-controlled, randomized, multi-centre, phase III studies. Median values of each laboratory parameters from baseline and after treatment (end of the double-blind treatment period) were compared. The three studies included a total of 550 adult patients (271 on placebo and 279 on active treatment) suffering from either birch or grass pollen allergic rhinoconjunctivitis with or without asthma.

**Table 1:** Hematology and blood chemistry laboratory parameters measured in the three phase III clinical studies

**Parameter (Unit)**	**Normal range**
**Hematology**	
Platelets [1000/μL]	150–400
Leukocytes [1000/μL]	5–10
Lymphocytes [%]	20–40
Basophils [%]	0.5–1
Eosinophils [%]	1–4
Monocytes [%]	2–8
Neutrophils [%]	55–70
Erythrocytes [10^6^/μL]	4.2–7.6
Haematocrit [%]	37–52
Haemoglobin [g/dL]	12–18
MCH [pg]^1^	28–33
MCHC [g/dL]^1^	33–36
MCV [fL]^1^	80–96
**Blood chemistry**	
Potassium [mmol/L]	3.5–5
Sodium [mmol/L]	136–145
Gamma-GT [U/L]^2^	8–38
SGOT(AST) [U/L]	0–35
SGPT(ALT) [U/L]	4–36
Creatinine [mg/dL]	0.5–1.2
Bilirubin [mg/dL]	0.3–1
Urea [mg/dL]	10–20

^1^N/A on Pre-seasonal SCIT 6-grass.

^2^N/A on SCIT rBirch.

Normal ranges modified from Kratz et al. [5].

The first study was performed with sublingual immunotherapy (SLIT) of the high dose grass pollen preparation (ALLERSLIT® forte) (SLIT 6-grass study) and included 185 patients [6,7]. Mean age of patients was 33.2 years (SD 10.3, range 17–59); 63% of patients were male, 37% female. The second trial evaluated subcutaneous immunotherapy (SCIT) with an aluminium hydroxide-adsorbed recombinant hypoallergenic variant of the major birch pollen allergen (rBet v1-FV) (SCIT rBirch study) and included 211 patients [8]. Mean age of patients was 38.2 years (SD 11.0, range 18–59); 47% of patients were male, 53% female. The third study assessed pre-seasonal SCIT of grass pollen allergoid (Allergovit®) (pre-seasonal SCIT 6-grass study) and included 154 patients [9]. Mean age of patients was 34.4 years (SD 10.6, range 18–60); 42% of patients were male, 58% female.

All patients had a history of IgE-mediated seasonal allergic rhinitis/conjunctivitis with or without asthma (GINA stage I and II) caused by grass pollen (grass studies) or birch pollen (birch study), positive skin prick test result to grass pollen extract (grass studies) or birch pollen extract (birch study), and positive conjunctival provocation test with grass pollen allergens (grass studies) or birch pollen allergens (birch study). Only patients with moderate to severe allergic symptoms to grass or birch pollen were included in the clinical trials. Patients were excluded if they previously had undergone grass or birch pollen immunotherapy or suffered from other seasonal allergies in the same period as the relevant grass or birch pollen, or had a history of cardiovascular or other immunological or medically relevant diseases.

After up-dosing patients in the SLIT 6-grass study were treated daily with high-dose sublingual 6-grass pollen preparation (*Holcus lanatus*, *Dactylis glomerata*, *Lolium perenne*, *Phleum pratense*, *Poa pratensis*, and *Festuca elatior*) or placebo from January 2004 until end of grass pollen season in 2005. The group 5 allergen content of the active maintenance dose corresponded to 40 μg. Patients in the SCIT rBirch study were treated with subcutaneous injections of recombinant hypoallergenic variant of the major birch pollen allergen (rBet v1-FV, maintenance dose 80 μg every 6 ± 2 weeks) or placebo from October 2004 until end of birch pollen season in 2006. Treatment of patients in the SCIT 6-grass study included pre-seasonal weekly injections with either 6-grass pollen (*Holcus lanatus, Dactylis glomerata, Lolium perenne, Phleum pratense, Poa pratensis, Festuca pratensis*) allergoid or placebo until the expected onset of the grass pollen season 2001 and 2002.

Written informed consent was obtained from the patients before they were enrolled in the studies, and the studies were performed in accordance with good clinical practice. The ethics committees of the participating countries approved the studies. A list of all participating ethics committees is included in Additional file 1. Matching placebo solutions were used as comparator in all three studies. All study preparations were manufactured by Allergopharma GmbH & Co. KG, Reinbek, Germany.

## Results

Demographic and baseline characteristics showed that the two treatment groups in all three studies were well matched in terms of baseline characteristics. Details of efficacy and safety outcome as well as specific immunological measures are described elsewhere [6,8,9].

**Figure 1:**
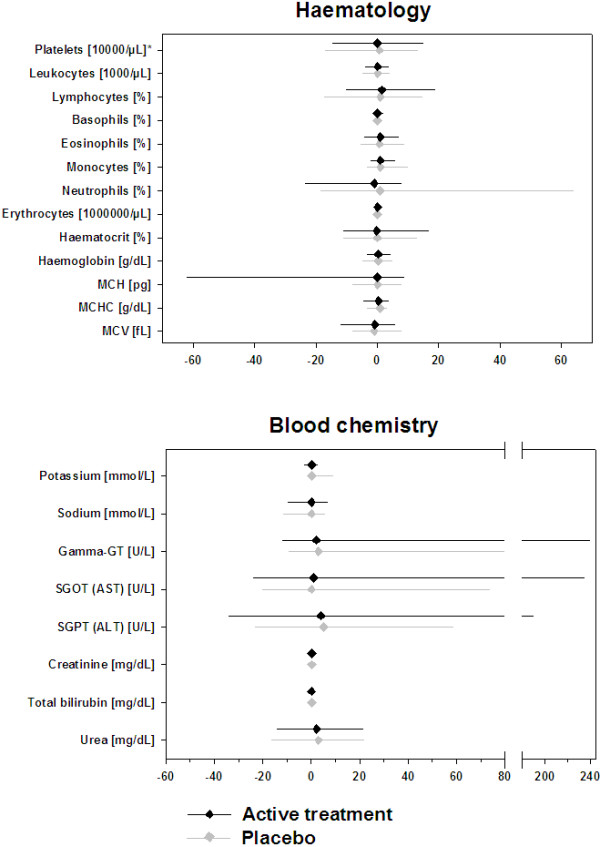
Absolute change from baseline to end of treatment for hematology (upper panel) and blood chemistry (lower panel) parameters (SLIT 6-grass: n = 91 for placebo and n = 94 for active treatment).

**Figure 2:**
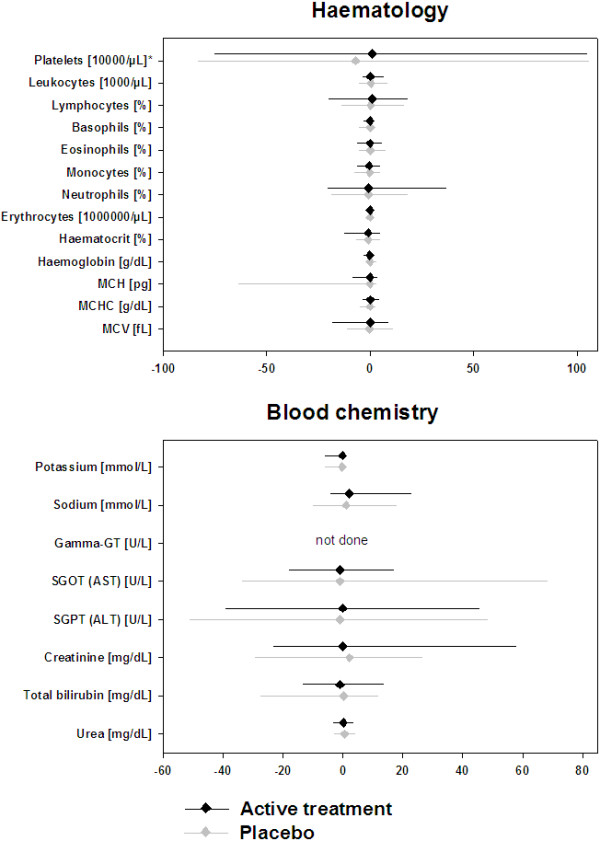
Absolute change from baseline to end of treatment for hematology (upper panel) and blood chemistry (lower panel) parameters (SCIT rBet v1-FV: n = 103 for placebo and n = 108 for active treatment).

**Figure 3:**
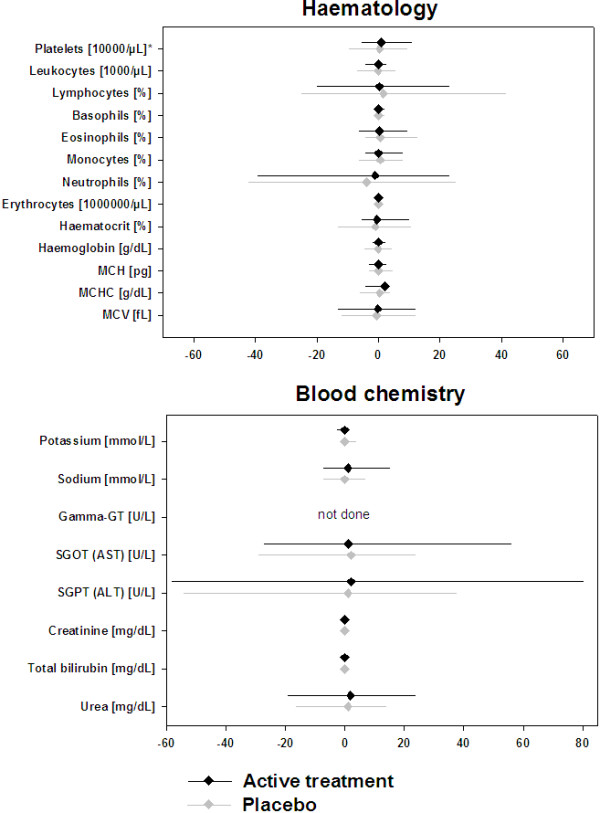
Absolute change from baseline to end of treatment for hematology (upper panel) and blood chemistry (lower panel) parameters (Pre-seasonal SCIT 6-grass: n = 77 for placebo and n = 77 for active treatment).

No differences were observed between placebo and active groups at baseline regarding hematology and clinical chemistry parameters. The baseline median values for all three studies are shown in Table 2.Allergen specific immunotherapy with either administration form or formulation did not show any influence on any of the 21 laboratory parameters analyzed (Figures 1, 2 and 3). The changes in median values were insignificant and there were no differences between placebo patients and actively treated patients in any of the studies.

**Table 2:** Baseline values for measured hematology and blood chemistry parameters (SLIT 6-grass: n = 91 for placebo and n = 94 for active treatment; SCIT rBet v1-FV: n = 103 for placebo and n = 108 for active treatment; pre-seasonal SCIT 6-grass: n = 77 for placebo and n = 77 for active treatment)

	**SLIT 6-grass**				**SCIT rBirch**				**Pre-seasonal SCIT 6-grass**			
**Parameter (unit)**	**Placebo**		**Active**		**Placebo**		**Active**		**Placebo**		**Active**	
**Hematology**	**Median**	**Range**	**Median**	**Range**	**Median**	**Range**	**Median**	**Range**	**Median**	**Range**	**Median**	**Range**
Platelets [1000/μL]	247.5	[134.0; 401.0]	249	[148.0; 393.0]	247	[149.0; 429.0]	236	[121.0; 369.0]	249	[100.0; 462.0]	261	[152.0; 377.0]
Leukocytes [1000/μL]	6.4	[3.2; 12.5]	6.3	[3.1; 14.6]	6	[3.3; 12.8]	5.8	[3.1; 12.1]	6.5	[3.7; 11.8]	6	[3.6; 14.6]
Lymphocytes [%]	26.3	[16.5; 42.7]	27	[14.6; 47.0]	31.4	[1.7; 61.0]	33.8	[8.8; 63.0]	29	[2.5; 52.7]	31.2	[2.7; 49.1]
Basophils [%]	0.6	[0.0; 1.9]	0.6	[0.0; 1.8]	0.6	[0.0; 2.1]	0.6	[0.0; 3.7]	0.5	[0.0; 1.8]	0.7	[0.0; 3.9]
Eosinophils [%]	1.9	[0.7; 16.1]	2	[0.0; 10.5]	2.4	[0.0; 11.1]	2.7	[0.7; 8.4]	2.3	[0.3; 10.3]	2.7	[0.1; 10.7]
Monocytes [%]	6.6	[0.3; 11.2]	6.2	[3.3; 12.2]	6.6	[2.6; 13.3]	7	[2.8; 17.0]	6.9	[0.5; 16.9]	6.9	[0.9; 15.7]
Neutrophils [%]	60	[1.0; 76.2]	60	[43.0; 77.4]	58	[30.0; 92.3]	55.7	[25.0; 86.0]	58.8	[34.0; 86.8]	56.6	[38.6; 72.8]
Erythrocytes [10^6^/μL]	4.8	[3.8; 6.0]	4.8	[4.0; 6.0]	4.8	[3.8; 5.8]	4.7	[3.6; 5.5]	4.62	[3.8; 5.8]	4.65	[3.9; 5.9]
Haematocrit [%]	44	[36.9; 52.0]	44.1	[34.0; 55.2]	42	[35.0; 51.0]	41.9	[33.0; 56.0]	42	[32; 50.0]	41.1	[26.9; 52.2]
Haemoglobin [g/dL]	14.7	[12.0; 18.0]	14.9	[12.0; 18.3]	14.4	[11.6; 16.8]	14	[10.5; 17.2]	14.1	[9.9; 17.4]	14.01	[8.0; 17.3]
MCH [pg]	29.5	[21.0; 33.1]	29.7	[21.0; 90.0]	30	[25.0; 33.8]	30.3	[23.0; 34.0]	ND	ND	ND	ND
MCHC [g/dL]	32.9	[30.3; 35.5]	33.3	[29.9; 36.5]	34	[29.1; 36.2]	33.6	[27.3; 36.9]	ND	ND	ND	ND
MCV [fL]	88.8	[83.0; 103.0]	89	[76.0; 106.0]	89	[76.0; 105.0]	90	[72.0; 108.0]	ND	ND	ND	ND
**Blood chemistry**												
Potassium [mmol/L]	4.2	[3.1; 5.4]	4.2	[3.7; 7.3]	4.2	[3.3; 6.2]	4.2	[3.1; 7.3]	4.8	[3.5; 10.0]	4.6	[3.7; 10.0]
Sodium [mmol/L]	140	[134.0; 148.4]	140	[134.0; 149.5]	140	[133.0; 147.0]	140	[127.0; 146.4]	139	[132.0; 139.0]	139.1	[133.0; 147.0]
Gamma-GT [U/L]	16	[7.0; 72.0]	19	[4.0; 243.0]	ND	ND	ND	ND	14	[5.0; 58.0]	19	[4.0; 190.2]
SGOT (AST) [U/L]	21	[6.0; 42.0]	23.5	[8.0; 62.0]	23	[8.0; 51.0]	25	[11.0; 72.0]	14.5	[5.0; 31.0]	15	[5.0; 36.0]
SGPT (ALT) [U/L]	20	[6.0; 56.0]	24	[7.0; 93.0]	22	[5.4; 84.0]	22	[6.0; 113.0]	18	[4.0; 18.0]	18.6	[5.0; 61.0]
Creatinine [mg/dL]	0.9	[0.5; 1.4]	0.9	[0.6; 1.3]	0.9	[0.5; 1.3]	0.8	[0.3; 1.3]	0.9	[0.6; 1.3]	0.9	[0.6; 1.4]
Bilirubin [mg/dL]	0.7	[0.2; 1.5]	0.7	[0.2; 3.1]	0.6	[0.1; 2.8]	0.5	[0.2; 2.5]	0.6	[0.2; 2.1]	0.6	[0.2; 2.7]
Urea [mg/dL]	29	[17.0; 53.0]	29	[19.0; 54.0]	28	[10.0; 47.0]	27	[10.0; 47.0]	26	[12.0; 52.0]	26	[6.4; 43.2]

ND = Not done.

Few patients had a change in laboratory parameters from within normal range at baseline to either below or above at end-of-treatment. No differences between active and placebo were seen with respect to number of patients with such a change. The numbers of analyses in the respective treatment groups which have changed to values outside the defined normal range are listed in Table 3. The laboratory parameters where most analyses showed a change were leukocytes, neutrophils, eosinophils, lymphocytes and monocytes; however no differences between active and placebo were observed.

**Table 3:** Number of patients with a change in hematology and blood chemistry parameter from normal at baseline to below or above the defined normal range at end of treatment

**Parameter (Unit)**	**Placebo (n = 271)**	**Active (n = 279)**
**Hematology**		
Platelets [1000/μL]	5	2
Leukocytes [1000/μL]	15	21
Lymphocytes [%]	32	26
Basophils [%]	8	13
Eosinophils [%]	26	33
Monocytes [%]	17	16
Neutrophils [%]	25	27
Erythrocytes [10^6^/μL]	17	13
Haematocrit [%]	17	13
Haemoglobin [g/dL]	15	13
MCH [pg]^1^	11	9
MCHC [g/dL]^1^	15	15
MCV [fL]^1^	9	9
**Blood chemistry**		
Potassium [mmol/L]	11	13
Sodium [mmol/L]	9	7
Gamma-GT [U/L]^2^	6	3
SGOT (AST) [U/L]	3	5
SGPT (ALT) [U/L]	10	8
Creatinine [mg/dL]	12	9
Bilirubin [mg/dL]	3	4
Urea [mg/dL]	4	7

^1^N/A on Pre-seasonal SCIT 6-grass.

^2^N/A on SCIT rBirch.

## Discussion

We selected thirteen hematology laboratory parameters and eight clinical chemistry parameters as a standard test battery in order to control that the different allergen formulations and routes of administration did not lead to pathophysiological changes in respective organs or blood.

We performed the tests prior to treatment and found that among patients selected for immunotherapy, the pathophysiology and the patient’s allergic disease history had not lead to abnormal values. Only a few analysis results were outliers in relation to the defined normal range of distribution in the standard population. Since the most important contraindication for allergen specific immunotherapy are cardiovascular and immunological diseases [10] it seems relevant to measure standard tests which could indicate other important disorders than allergy in the individual patient initially recruited. Other laboratory tests of specific interest in relation to allergen specific immunotherapy are measures of specific antibodies such as IgE and IgG4 [11]. These antibodies are directly related to the pathophysiology of respiratory allergic diseases and the immunological changes in these antibodies following treatment is related to the clinical effects [12,13]. These antibodies should usually be measured during investigations of allergen specific immunotherapy.

We selected three different immunotherapy studies, all performed in adults with the purpose of following the basic laboratory parameters during treatment. In all these studies we found an increase in the induction of specific antibodies [6,9,14] but allergen specific immunotherapy treatment did not in any case result in significant changes in the basic clinical chemistry and hematology parameters. The three studies represented three different approaches for allergen specific immunotherapy. In principle the inclusion criteria were the same in all three studies and the treatment period was up to 24 months. One study was performed with a standard pre-seasonal grass pollen allergen extract given as weekly injections for two consecutive pre-seasonal periods and another study performed with sublingual treatment including daily self-administration for two years. The last study included was performed with a recombinant major allergen vaccine for birch allergy (rBet v1-FV). Independently of administration route and formulation we found no significant changes in basic blood chemistry or hematology measures during the study period. We conclude that the allergen specific immunotherapy treatment as investigated did not lead to abnormal changes in basic immunological and metabolic processes. This is in accordance with an earlier publication [15]. Since the active drugs in this case are natural occurring protein substances or well defined recombinant proteins there are also no scientific rationale for expecting major changes in the parameters we investigated except the induction of specific antibodies.

## Conclusion

The Guidelines from EMA on safety pharmacology studies conclude that the basic pharmacology core battery for investigation of vital functions based on scientific rationale can in some situations be saved [1]. With this paper we confirm such a scientific rationale and conclude that the evaluation of these parameters is relevant for inclusion of patients during the screening process. This study with different preparations and routes of application indicates that the value of repeated measurements of standard clinical chemistry and hematology parameters during allergen immunotherapy should be discussed further.

## Abbreviations

EMA: European medical agency; Pre-seasonal SCIT 6-grass study: Subcutaneous immunotherapy study with pre-seasonal 6-grass pollen allergoid; SCIT: Subcutaneous immunotherapy; SCIT rBirch study: Subcutaneous immunotherapy study with an aluminium hydroxide-adsorbed recombinant hypoallergenic folding variant of the major birch pollen allergen (rBet v1-FV); SLIT: Sublingual immunotherapy; SLIT 6-grass study: Sublingual immunotherapy study with a high dose 6-grass pollen preparation.

## Competing interests

All authors are employees of Allergopharma GmbH & Co. KG. The authors declare that they have no competing interests.

## Authors’ contributions

DH, VG and AN were involved in the design of the studies, data interpretation and drafting, revising and final approval of the manuscript. All authors read and approved the final manuscript.

## Supplementary Material

Additional file 1 A list of all participating ethics committees. Click here for file
