# Supplementary material for: Allergen specific immunotherapy has no influence on standard chemistry and hematology laboratory parameters in clinical studies
Source: Clin Transl Allergy. 2014 May 22;4:18. doi: 10.1186/2045-7022-4-18 (PMC4064516; doi:10.1186/2045-7022-4-18)
Supplement: Additional file 1 — A list of all participating ethics committees. [file 2045-7022-4-18-S1.docx]

| SLIT 6-grass study | SCIT rBirch study | SCIT 6-grass study |
| --- | --- | --- |
| Ethik-Kommissionen bei der Ärztekammer Schleswig-Holstein  Bismarckallee 8 – 12 23795 Bad Segeberg | Ethik-Kommissionen bei der Ärztekammer Schleswig-Holstein  Bismarckallee 8 – 12 23795 Bad Segeberg | Ethik-Kommissionen bei der Ärztekammer Schleswig-Holstein  Bismarckallee 8 – 12 23795 Bad Segeberg |
| Ethik-Kommission der Ärztekammer Hamburg Weidestraße 122 b  22083 Hamburg | Ethik-Kommission der Ärztekammer Hamburg Weidestraße 122 b  22083 Hamburg | Landesärztekammer Brandenburg Ethikkommission  Dreifertstraße 12  03044 Cottbus |
| Ethik-Kommission der Ärztekammer Westfalen-Lippe und der Medizinischen Fakultät der Westfälischen Wilhelms-Universität Münster  Gartenstraße 210 - 214 48147 Münster | Ethik-Kommission der sächsischen Landesärztekammer Schützenhöhe 16 01099 Dresden | Ethik-Kommission der sächsischen Landesärztekammer Schützenhöhe 16 01099 Dresden |
| Ethik-Kommission der Landesärztekammer in Hessen Im Vogelgesang 3 60488 Frankfurt | Ethik-Kommission der Landesärztekammer in Hessen Im Vogelgesang 3 60488 Frankfurt | Ethik-Kommission der Landesärztekammer in Hessen Im Vogelgesang 3 60488 Frankfurt |
| Ethik-Kommission der  Friedrich-Schiller-Universität Jena Postfach Bachstraße 18 07740 Jena | Ethik-Kommission der  Friedrich-Schiller-Universität Jena Postfach Bachstraße 18 07740 Jena | Ethik-Kommission der  Friedrich-Schiller-Universität Jena Postfach Bachstraße 18 07740 Jena |
| Ethik-Kommission des Landes Berlin Landesamt für Gesundheit und Soziales Fehrbelliner Platz 1 10707 Berlin | Ethik-Kommission des Landes Berlin Landesamt für Gesundheit und Soziales Fehrbelliner Platz 1 10707 Berlin | Ethik-Kommission der Ärztekammer Westfalen-Lippe und der Medizinischen Fakultät der Westfälischen Wilhelms-Universität Münster  Gartenstraße 210 - 214 48147 Münster |
| Ethikkommission der Med. Fakultät der HHU Düsseldorf Kinderklinik, Geb. 13.41 Moorenstr. 5 40225 Düsseldorf | Ethikkommission der Med. Fakultät der HHU Düsseldorf Kinderklinik, Geb. 13.41 Moorenstr. 5 40225 Düsseldorf | Guy's Research Ethics Committee London SE1 9RT |
| Ethik-Kommission bei der  Ärztekammer Niedersachsen Berliner Allee 20 30175 Hannover | Ethik-Kommission bei der  Ärztekammer Niedersachsen Berliner Allee 20 30175 Hannover |  |
| Ethik-Kommission der Ärztekammer Nordrhein Tersteegenstraße 9  40474 Düsseldorf | Geschäftsstelle Ethikkommission Universität zu Köln Gebäude 5, Etage 2a, Raum 015 Kerpener Str. 62 50937 Köln |  |
| Med. Ethikkommission II der  Medizinische Fakultät Mannheim  der Universität Heidelberg Maybachstr. 14  68169 Mannheim | Ethik-Kommission der Ärztekammer Westfalen-Lippe und der Medizinischen Fakultät der Westfälischen Wilhelms-Universität Münster  Gartenstraße 210 - 214 48147 Münster |  |
| Ethik-Kommission der Universität Ulm Helmholtzstraße 20  89081 Ulm | Ethik-Kommission der Medizinischen Fakultät  Friedrich-Alexander-Universität Erlangen-Nürnberg Krankenhausstr. 12 91054 Erlangen |  |
| Ethik-Kommission der Bayerischen Landesärztekammer Mühlbaurstraße 16 81677 München | Komisji Bioetycznej Uniwersytet Medyczny  Al. Kościuszki 4 90-419 Łódź |  |
| Ethik Committee for Medical Investigations Faculty of Medicine "50 Divizija" br. 6  1000 Skopje Mazedonien | KOMISJA BIOETYCZNA przy DOLNOŚLĄSKIEJ IZBIE LEKARSKIEJ ul. Kazimierza Wielkiego 45 50-077 Wrocław |  |
| Komisji Bioetycznej Warszawski Uniwersytet Medyczny ul. Żwirki i Wigury 61 02-091 Warszawa | Komisji Bioetycznej Uniwersytet Medyczny  Al. Kościuszki 4 90-419 Łódź |  |
| Komisji Bioetycznej Uniwersytet Medyczny  Al. Kościuszki 4 90-419 Łódź | Okręgowa Izba Lekarska ul. Czerwona 3 93-005 Łódź |  |
